# Denial of pregnancy: a review

**DOI:** 10.1192/j.eurpsy.2022.2226

**Published:** 2022-09-01

**Authors:** M.D.C. Molina Liétor, I. Cuevas Iñiguez

**Affiliations:** 1 Hospital Universitario Príncipe de Asturias, Psiquiatría, Alcalá de Henares, Spain; 2 Hospital Principe de Asturias, Psiquiatría, Alcala de Henares, Spain

**Keywords:** Pregnancy, denial

## Abstract

**Introduction:**

Denial of pregnancy is a condition that the pregnant woman is not aware that she is pregnant. It appears in one in every five hundred pregnancies, approximately. Women who present denial of pregnancy do not usually present comorbidity with another psychiatric pathology, although dependent personality traits, low self-esteem, loneliness and poor communication with the partner have been described as features among patients.

**Objectives:**

The objective of this work is to present the current information on the denial of pregnancy.

**Methods:**

A review about denial of pregnancy.

**Results:**

Denial of pregnancy can be classified as psychotic denial (the woman may misinterpret the symptoms and physical changes of pregnancy, usually in strange ways. These people do not hide their pregnancy and those around them are often aware of the situation) or non-psychotic (the patient has the judgment of reality preserved). Non-psychotic denial can be affective: (the woman intellectually recognizes that she is pregnant but does not experience the emotional or behavioral changes that usually occur. This type of denial is related to feelings of detachment from the baby) or generalized (occurs when the The woman not only does not suffer the emotional changes of pregnancy, but also does not know the existence of pregnancy itself. Weight gain, amenorrhea and other changes inherent to this state may not be present or be misinterpreted. It may be that neither the family nor the environment realizes the pregnancy and then there is a collective denial of the pregnancy.)

**Conclusions:**

Research and prevention of perinatal pathology should be a priority.

**Disclosure:**

No significant relationships.

